# Response to SARS-CoV-2 vaccines in patients receiving B-cell modulating antibodies for renal autoimmune disease

**DOI:** 10.1186/s12879-022-07722-7

**Published:** 2022-09-14

**Authors:** Frederic Arnold, Daniela Huzly, Yakup Tanriver, Thomas Welte

**Affiliations:** 1 Department of Medicine IV, Medical Center, Faculty of Medicine, University of Freiburg, Hugstetter Strasse 55, 79106 Freiburg, Freiburg, Germany; 2 Institute for Microbiology and Hygiene, Medical Center, Faculty of Medicine, University of Freiburg, Freiburg, Germany; 3 Institute for Virology, Medical Center, Faculty of Medicine, University of Freiburg, Freiburg, Germany; 4 Friedrich Miescher Institute for Biomedical Research, Basel, Switzerland

**Keywords:** COVID-19, SARS-CoV-2, CD20, Rituximab, Kidney

## Abstract

**Background:**

Effective SARS-CoV-2 vaccination in patients receiving treatment with B-cell depleting agents is challenging. Information on vaccination responses in these patients are a valuable tool to develop efficient vaccination regimens.

**Methods:**

In this single-center retrospective observational study, we report the humoral and cellular response in 34 patients receiving anti-CD20 antibody treatment for renal immune disease.

**Results:**

After base immunization with SARS-CoV-2-vaccines, 92.0% developed a cellular, 32.4% a humoral response. Humoral immunity correlated with B-cell counts and the timespan between anti-CD20 antibody treatment and vaccination. All patients with > 21/µl B-cells, or > 197 days after treatment showed humoral response.

**Conclusions:**

Adequate timing of SARS-CoV-2-vaccinations after anti-CD20 antibody treatment and CD19 measurements are crucial to generate immunity. Awaiting partial B-cell recovery by postponing regularly scheduled anti-CD20 treatment should be considered in patients with stable immune disease.

*Trial registration:* This study has been retrospectively registered in the German Clinical Trials Register (DRKS00027049) on 29/10/2021.

**Supplementary Information:**

The online version contains supplementary material available at 10.1186/s12879-022-07722-7.

## Background

Successful vaccination of risk groups is essential to reduce morbidity and mortality in the COVID-19 (coronavirus disease 2019) pandemic. Vaccine-induced immunity relies on antibody-driven (humoral), and T-cell mediated (cellular) immune response [1]. Antibodies targeting the viral spike protein contribute to virus neutralization and can prevent the entry of SARS-CoV-2 into host cells [2, 3].

Effective vaccination of immunocompromised patients is especially challenging as many immunosuppressive agents impair the response to vaccines, increasing the risk for severe COVID-19, prolonged infection, and viral evolution [4, 5].

Several immune-mediated and hematologic diseases are treated with monoclonal anti-CD20 antibodies. These drugs deplete circulating B-cells, leading to impaired maturation of memory B-cells and reduced numbers of antibody-producing plasma cells[6]. Consequently, anti-CD20-treatment is associated with a compromised response to SARS-CoV-2-vaccines [7, 8].

Chronic kidney disease is a further independent risk factor known to impair vaccination responses [9]. Hence, vaccination of patients receiving B-cell depleting drugs for autoimmune renal diseases poses a huge challenge.

Here, we report the humoral and cellular immune response to mRNA- and vector-based SARS-CoV-2 vaccines (mRNA-1273, BNT162b2, ChAdOx1, and Ad26.COV2.S) in patients receiving the monoclonal anti-CD20 antibody Rituximab for renal autoimmune disease. Our results help to optimize vaccination strategies by taking prior therapies and cellular parameters into account.

## Methods

In this single-center retrospective observational study, we report data from the University of Freiburg Medical Center, Germany. Inclusion criteria were age ≥ 18 years, current treatment with the anti-CD20 antibody Rituximab for renal autoimmune disease, and completed base-immunization with either mRNA-based (BNT162b2, mRNA-1273), or vector-based (ChAdOx1, Ad26.COV2.S) SARS-CoV-2 vaccines. In vaccination regimens requiring two doses for base immunization, the second dose was given between 2 and 12 weeks after the first vaccination. Exclusion criteria were prior SARS-CoV-2 infections, and administration of antimetabolite drugs (Mycophenolate Mofetil, Azathioprine, Methotrexate) within two months before vaccination.

Demographic data, past medical history, treatment details and laboratory findings (estimated glomerular filtration rates [eGFR], proteinuria and hematuria) were extracted from electronic patient records of outpatient visits between April and September 2021. B cell counts were measured in between 5 weeks prior, to 12 weeks after vaccination by flow cytometry (details, see Additional file 1). In patients, where anti-CD20 antibody treatment was dated back longer than 12 months, the last available B-cell count after repopulation was used for analysis. SARS-CoV-2 specific S1-IgGs and S1-antigen T-cell IFN-γ response were quantified between 8 and 21 days after complete vaccination (details, see Additional file 1).

Positive humoral response was defined as anti-SARS-CoV-2-S1 IgG ≥ 21.8 BAU/ml according to manufacturers’ instructions. Positive cellular response was defined as IFN-γ-release to SARS-CoV-2 S1 antigen of ≥ 135 mIU/ml (for IFN-γ negative control < 100 mIU/ml), and ≥ 200 mIU/ml (for IFN-γ negative control > 100 mIU/ml), using a double-cut-off strategy integrating the result of background stimulation according to in-house standard procedure ([10]; details, see Additional file 1).

IFN-γ assays were not valid, or not available for 9 patients. Data for these patients were excluded from the analysis of cellular response.

Statistical analysis and data visualization were performed using R 4.1.0 statistical software. The Wilcoxon-Mann–Whitney test was used to calculate p-values. A two-sided α ≤ 0.05 was considered statistically significant. In log_2_-transformed plots of anti-SARS-CoV-2-S1 IgG titers and CD19 counts, a pseudo count of one was added to the data to retain zero values.

## Results

General characteristics of the study population are summarized in Additional file 1: Table S1. 34 Caucasian, fully-vaccinated patients were included in the study (15 female [44.1%], 19 male [55.9%]). Patients received treatment with anti-CD20 antibody for Antineutrophil Cytoplasmic Antibody (ANCA)-associated vasculitis (AAV; n = 22 [64.7%]), Focal Segmental Glomerulosclerosis (FSGS; n = 1 [2.9%]), Membranous Glomerulopathy (MGN; n = 3 [8.8%]), Minimal Change Disease (MC; n = 3 [8.8%]), Goodpasture Disease (n = 4 [11.8%]), and Thrombotic Microangiopathy (TMA; n = 4 [11.8%]).

Anti-CD20 treatment was either administered every 6 months as part of a standard AAV-treatment regimen [11], or prescribed individually for other diagnoses. 18 patients (52.9%) received additional treatment with prednisone (2.5–10 mg/d). One patient received additional therapy with hydroxychloroquine (200 mg/d) for Systemic lupus erythematodes (SLE).

26 patients (76.5%) received mRNA vaccines (BNT162b2: n = 25 [73.5%]), mRNA-1273: n = 1 [2.9%]). Six patients (17.6%) received vector-based vaccines (ChAdOx1: n = 3 [8.8%], Ad26.COV2.S: n = 3 [8.8%]). Two patients (5.9%) received a first dose of the vector-based vaccine ChAdOx1, followed by a mRNA-based vaccine (BNT162b2: n = 1 [2.9%]; mRNA-1273: n = 1 [2.9%]).

Humoral response was detected in 11 (32.4%) patients. Cellular response was detected in 23 (92%) patients. One individual had neither response, while an isolated response was observed in another individual. Kidney function, proteinuria, or hematuria, as well as timespans between first and second vaccinations and the timepoint of testing after vaccination were not altered between response groups (Table 1).

**Table 1:** Humoral and cellular vaccination response

	Humoral			Cellular		
	No response	Response	P	No response	Response	p
Patients, n (%)	21 (61.8)	13 (38.2)		2 (8.0)	23 (92.0)	
Female, n (%)	8 (38.1)	7 (54.8)		0 (0.0)	9 (39.1)	
Mean Age at vaccination (SD)	62.6 (15.7)	53.5 (18.5)	0.07	66.0 (22.6)	55.7 (16.9)	^†^
Cause for Immunosuppression						
AAV, n (%)	18 (85.7)	4 (30.8)		2 (100.0)	12 (52.2)	
FSGS, n (%)	1 (4.8)	0 (0.0)		0 (0.0)	1 (4.3)	
MGN, n (%)	1 (4.8)	2 (15.4)		0 (0.0)	2 (8.7)	
MC, n (%)	1 (4.8)	2 (15.4)		0 (0.0)	3 (13.0)	
TMA, n (%)	0 (0.0)	4 (30.8)		0 (0.0)	4 (17.4)	
M. Goodpasture, n (%)	0 (0.0)	1 (7.7)		0 (0.0)	1 (4.3)	
Current immunosuppression						
Anti-CD20 antibody						
Regular regimen, n (%)	17 (81.0)	3 (23.1)		2 (100.0)	12 (52.2)	
Irregular regimen, n (%)	4 (19.0)	10 (76.9)		0 (0.0)	11 (47.8)	
Steroid, n (%)	12 (57.1)	1 (7.7)		2 (100.0)	6 (26.1)	
Hydroxychloroquine, n (%)	0 (0.0)	1 (7.7)		1 (50.0)	0 (0.0)	
Previous immunosuppression						
Cyclophosphamide, n (%)	13 (61.9)	5 (38.5)		2 (100.0)	9 (39.1)	
High-dose Steroid, n (%)	20 (95.2)	13 (100.0)		2 (100.0)	22 (95.7)	
Mycophenolate Mofetil, n (%)	5 (23.8)	3 (23.1)		0 (0.0)	8 (34.8)	
Azathioprine, n (%)	1 (4.8)	1 (7.7)		0 (0.0)	2 (8.7)	
Cyclosporine A, n(%)	5 (23.8)	3 (23.1)		0 (0.0)	8 (34.8)	
Leflunomide, n (%)	1 (4.7)	0 (0.0)		0 (0.0)	1 (4.3)	
Metotrexate, n (%)	1 (4.3)	0 (0.0)		0 (0.0)	1 (4.3)	
Kidney function						
eGFR, CKD-EPI; ml/min/1.73m^2^ (SD)	47.4 (25.5)	63.2 (26.9)	0.11	53.5 (30.4)	60.2 (24.0)	^†^
Proteinuria, g/g (SD)	1.2 (3.0)	1.0 (1.9)	0.30	0.5 (0.6)	1.2 (3.0)	^†^
Hematuria, Stix (SD)	1.4 (1.3)	0.7 (0.8)	0.15	0.5 (0.7)	1.1 (1.2)	^†^
Vaccines used						
2 × BNT162b2, n (%)	16 (76.2)	9 (69.2)		2 (100.0)	15 (65.2)	
2 × mRNA-1273, n (%)	1 (4.8)	0 (0.0)		0 (0.0)	1 (4.3)	
2 × ChAdOx1, n (%)	1 (4.8)	2 (15.4)		0 (0.0)	3 (13.0)	
1 × Ad26.COV2.S, n (%)	2 (9.5)	1 (7.7)		0 (0.0)	2 (8.7)	
1 × ChAdOx1, 1 × BNT162b2, n (%)	1 (4.7)	0 (0.0)		0 (0.0)	1 (4.3)	
1 × ChAdOx1, 1 × mRNA-1273, n (%)	0 (0.0)	1 (7.7)		0 (0.0)	1 (4.3)	
Vaccination characteristics						
Days anti-CD20 antibody to 1st vaccination (SD)	111.2 (51.7)	553.5 (553.8)	< 0.01	131.0 (50.9)	327.4 (453.5)	^†^
CD19 counts at vaccination, cells/µl (SD)	1.2 (4.6)	146.1 (241.1)	< 0.01	0.5 (0.7)	74.4 (191.9)	^†^
Days between 1st and 2nd vaccine (SD)	44.8 (17.6)	42.3 (22.2)	0.26	30.0 (7.1)	46.6 (21.0)	^†^
Days full vaccination to laboratory analysis (SD)	35.7 (29.6)	46.6 (36.7)	0.30	83.0 (49.5)	40.0 (32.9)	^†^

P-values were calculated using Wilcoxon-Mann–Whitney test. ^†^P-values not reported, as n = 2 in cellular no-response group. *AAV* ANCA-associates vasculitis, *FSGS* Focal segmental glomerulosclerosis, *MGN* Membranous glomerulopathy, *MC* Minimal Change disease, *SD* Standard deviation, *TMA* Thrombotic microangiopathy

Kidney function, proteinuria and hematuria did not correlate with SARS-CoV-2-IgGs or IFN-γ-release (Additional file 1: Fig. S1A). Mean age, anti-SARS-CoV-2-S1 IgG levels and IFN-γ-release were not statistically significant between sexes (Additional file 1Table S2).

**Fig. 1:**
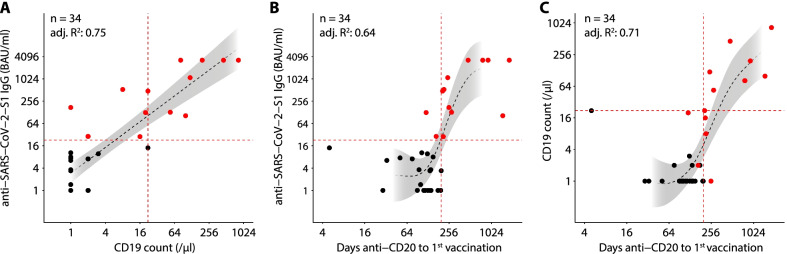
SARS-CoV-2 Vaccination response correlates with CD19 cell count at vaccination, and with the interval size between Rituximab treatment and vaccination. **A** Scatter plot showing correlation between CD19 counts and anti- SARS-CoV-2-S1 IgGs. A linear regression model was fitted to the data. **B** Scatter plot showing correlation between days between Rituximab to 1^st^ vaccination and anti-SARS-CoV-2-S1 IgGs. **C** Scatter plot showing correlation between days between Rituximab to 1^st^ vaccination and CD19 counts. In (**B**), and (**C**), a generalized additive regression model was fitted to the data. In **A-C**, patients with positive humoral response are plotted in red, patients without humoral response are plotted in black. Black dashed lines indicate regression models fitted to the data. Grey areas indicate 95% confidence interval of the regression models. Dashed red lines indicate the cutoff for positive humoral response (≥ 21.8 BAU/ml), CD19 counts (> 21/µl), and days from anti-CD19-treatment to 1^st^ vaccination (> 197), above which humoral response was recorded for all patients, respectively

B-cell counts at vaccination correlated with the time from last anti-CD20-antibody treatment to vaccination and were significantly higher in patients with a humoral response (Table 1, Additional file 1: Fig. S1A). Further analysis showed a linear correlation between B-cell (CD19) counts and anti-SARS-CoV-2-S1 IgGs (Fig. 1A). Sigmoidal correlations were found between the time after the last anti-CD20 treatment and anti-SARS-CoV-2-S1 IgG levels, as well as CD19 counts (Fig. 1B–C). All patients with CD19 counts > 21/µl, or a > 197-day interval between anti-CD20 treatment and the first vaccination had a humoral vaccination response. Accordingly, patients receiving regular anti-CD20 treatment (every 6 months) had significantly lower CD19 counts and anti-SARS-CoV-2-S1 IgG levels (Additional file 1: Fig. S1B, C, Table S3), and were thus underrepresented in the humoral response group (9.1% in responders vs. 82.6% in non-responders, Table 1). Of note regular anti-CD20 treatment was overrepresented in AAV-patients (Additional file 1: Table S3), and these patients were significantly older (Additional file 1: Table S3, Fig. S1D). This is likely due to an overrepresentation of AAV-associated vasculitis in older patients (Additional file 1: Table S4, Fig. S1E [12],).

Patients receiving additional corticosteroids are overrepresented in humoral and cellular non-responder groups (Table 1). A subgroup-analysis of patients receiving regular anti-CD20 treatment revealed slightly higher CD19 counts, anti-SARS-CoV-2 IgGs, and significantly increased IFN-γ-release in patients without additional corticosteroid treatment (Additional file 1: Table S5, Fig. S1F).

Finally, there were no significant differences in humoral, or cellular response in patients receiving different vaccination regimens. However, in line with previous reports [13], we observed a trend towards increased IFN-γ-release in the two patients receiving a mixed vaccination regimen compared to other vaccination regimens (Additional file 1: Fig. S1G, H).

## Discussion

Anti-CD20 treatment impairs humoral response to vaccinations. Data on SARS-CoV-2 vaccines is still limited, but previous studies have shown negative effects on antibody production after the BNT162b2 vaccine in patients receiving B-cell depleting agents [7, 14].

This study confirms impaired humoral response to SARS-CoV-2 vaccinations in patients receiving anti-CD20 treatment for renal autoimmune diseases. We show that the humoral response strongly correlates with B-cell counts and the interval between last anti-CD20 antibody administration and vaccination. Both, time from Rituximab to humoral response and CD19 counts necessary for vaccination response, are similar to results reported in parallel studies [15–20]. This provides crucial information on how to time anti-CD20 antibody application and SARS-CoV-2 vaccinations.

Our study shows that the cellular response to SARS-CoV-2 vaccines is independent of CD19 depletion, confirming other recent reports [17, 21]. The extent of the protective effect of T cell immunity against COVID-19 should be addressed in future studies.

In line with other recent studies [22–24], we observed a trend for stronger cellular immune response in mixed vaccination regimens (first vector-based, then mRNA-based vaccine). Hence, a mixed vaccination regimen might be beneficial in patients receiving anti-CD20 treatment.

We do not observe reduced kidney function to be a statistically significant risk factor for impaired vaccination response. However, parallel studies report reduced response to mRNA SARS-CoV-2 vaccines in both subjects with chronic and end stage renal disease [25, 26].

This study has several limitations, the major being the small study population, the retrospective character, and the lack of a standardized follow-up. The multitude of anti-CD20 antibody treatment regimens further complicate the interpretation. However, our study provides valuable information on how to optimize the humoral vaccination response, and how to reduce the risk for COVID-19 in an especially vulnerable patient cohort.

## Conclusions

Based on our data, awaiting partial B-cell recovery, and thus postponing regularly scheduled anti-CD20 treatment, should be considered in patients with stable renal disease. We and others [27] observed impaired humoral and cellular immune responses in patients receiving steroid treatment; thus, vaccinations should be performed with minimal steroid therapy.

## Supplementary Information

**Additional file 1.** Supplemental Figure S1, Supplemental Tables S1–S5, Supplemental Methods.

## Data Availability

The datasets analyzed in the current study are not publicly available to ensure privacy of research participants and comply with regulations of the ethics approval. Fully anonymized raw datasets analyzed in this study are available from the corresponding author on reasonable request.

## Abbreviations

95% CI: 95% Confidence interval
AAV: ANCA-associated vasculitis
ANCA: Antineutrophil Cytoplasmic Antibody
BAU: Binding antibody units
CD: Cluster of differentiation
CKD: Chronic kidney disease
COVID-19: Coronavirus disease 2019
eGFR: Estimated glomerular filtration rate
IFN-γ: Interferon gamma
IgG: Immunoglobin G
MC: Minimal change disease
MGN: Membranous glomerulonephritis
SARS-CoV-2: Severe acute respiratory syndrome coronavirus type 2
SCr: Serum creatinine
SD: Standard deviation
SLE: Systemic lupus erythematodes
TMA: Thrombotic microangiopathy
