# Supplementary material for: Response to SARS-CoV-2 vaccines in patients receiving B-cell modulating antibodies for renal autoimmune disease
Source: BMC Infect Dis. 2022 Sep 14;22:734. doi: 10.1186/s12879-022-07722-7 (PMC9472190; doi:10.1186/s12879-022-07722-7)
Supplement: Supplementary file 1 — Additional file 1. Supplemental Figure S1, Supplemental Tables S1–S5, Supplemental Methods. [file 12879_2022_7722_MOESM1_ESM.pdf]

Response to SARS-CoV-2 vaccines in patients receiving B-cell modulating antibodies for renal autoimmune disease

Frederic Arnold, Daniela Huzly, Yakup Tanriver, Thomas Welte

Supplemental Material

Supplemental Figure S1

Supplemental Tables S1 - S5

Supplemental Methods

Supplemental References

Supplemental Figure

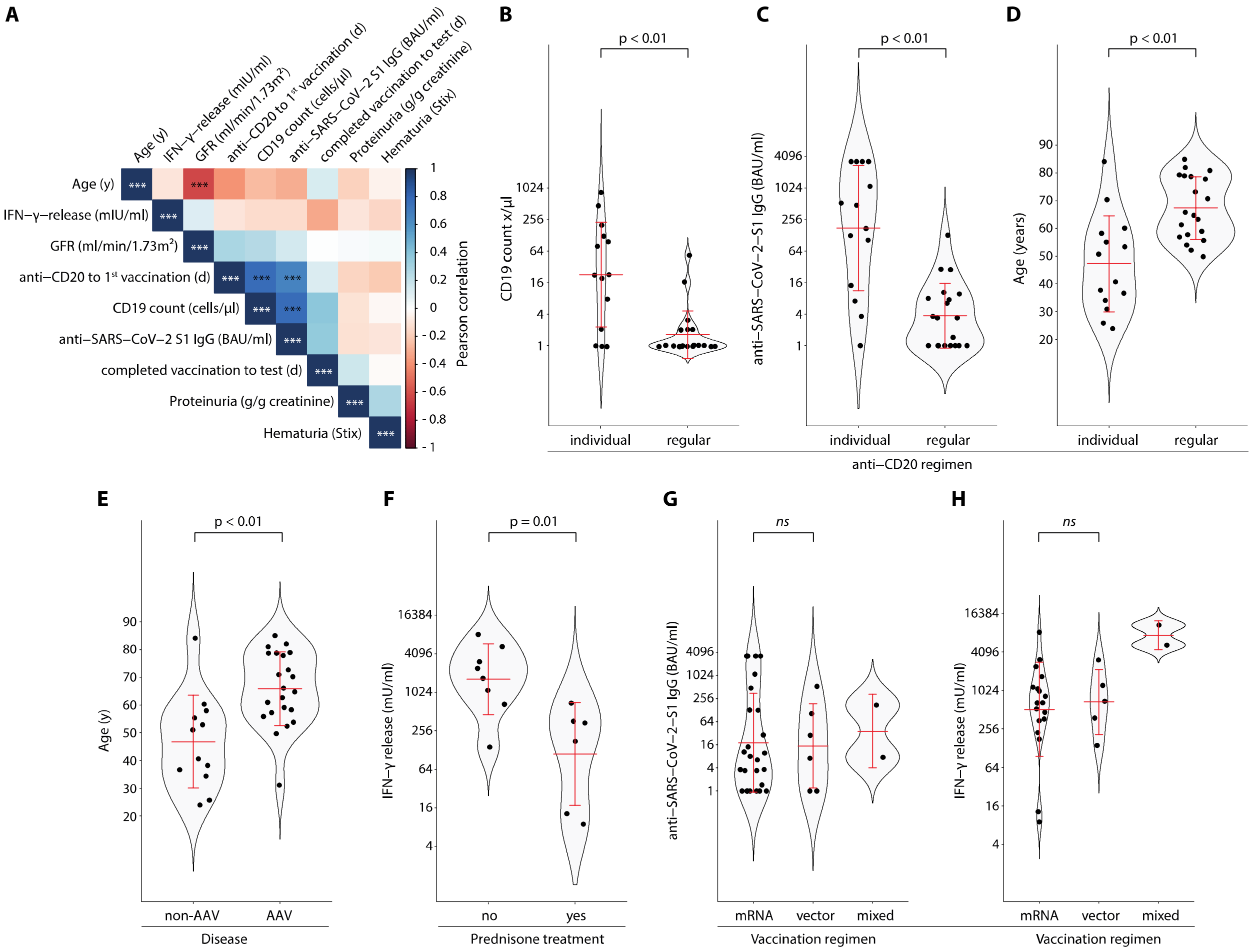

Supplemental Figure S1. (**A**) Heatmap showing Pearson correlation between numerical variables recorded in this study. Features are ordered by hierarchical clustering. For each pair of features, a significance test producing p-values and 95% confidence intervals was performed. *** p-value ≤ 0.0001 (**B-D**) Violin plots showing higher CD19-count, anti-SARS-CoV-s-S1 IgGs, and patient age in individual versus regular anti-CD20 regimen. (**E**) Violin plot showing higher patient age in AAV vs no AAV. (**F**) Violin plot showing significantly lower cellular vaccination response in patients treated with Prednisone. (**G, H**) Violin plots showing no significant differences of humoral (**G**) or cellular (**H**) vaccination responses in patients receiving mixed (first vector-based, then mRNA) vs mRNA, or vector-based vaccine, but a trend towards higher IFN-γ release in patients receiving a mixed vaccination regimen (n=2). P-values are calculated using Wilcoxon-Mann-Whitney test (**B-H**). For the comparison with the mixed vaccination regimen (n=2) no p-values are reported (**G, H**). Red lines in (**B-H**) indicate mean and standard deviation (SD). *ns* p-value > 0.05.

Supplemental Tables

	Study population
Patients, n	34
Female, n (%)	15 (44.1)
Mean Age at vaccination, years (SD)	59.1 (17.1)
**Cause for Immunosuppression**	
AAV, n (%)	22 (64.7)
FSGS, n (%)	1 (2.9)
MGN, n (%)	3 (8.8)
MC, n (%)	3 (8.8)
TMA, n (%)	4 (11.8)
M. Goodpasture, n (%)	1 (2.9)
**Current immunosuppressive treatment**	
Anti-CD20 antibody	
2x 1000 mg, 500 mg/6 months, n (%)	19 (55.9)
4x 375 mg/m2, 500 mg/6 months, n (%)	1 (2.9)
other regimen, n (%)	14 (41.2)
Steroid, n (%)	13 (38.2)
Hydroxychloroquine, n (%)	1 (2.9)
**Previous immunosuppressive treatment**	
Cyclophosphamide, n (%)	18 (52.9)
High-dose Steroid, n (%)	33 (97.1)
Mycophenolate Mofetil, n (%)	8 (23.5)
Azathioprine, n (%)	2 (5.9)
Cyclosporine A, n (%)	8 (23.5)
Leflunomide, n (%)	1 (2.9)
Metotrexate, n (%)	1 (2.9)
**Kidney function**	
eGFR – CKD-EPI, ml/min/1.73m^2 (SD)	53.4 (26.8)
Proteinuria, g/g Creatinine (SD)	1.2 (2.6)
Hematuria, Stix (SD)	1.1 (1.2)
**Vaccines used**	
2x BNT162b2, n (%)	25 (73.5)
2x mRNA-1273, n (%)	1 (2.9)
2x ChAdOx1, n (%)	3 (8.8)
1x Ad26.COV2.S, n (%)	3 (8.8)
1x ChAdOx1 + 1x BNT162b2, n (%)	1 (2.9)
1x ChAdOx1 + 1x mRNA-1273, n (%)	1 (2.9)

Supplemental Table S1. Base characteristics of the study population. Abbreviations: AAV, ANCA-associated vasculitis; FSGS, focal segmental glomerulosclerosis; MGN, membranous glomerulonephritis; MC, minimal change disease; TMA, thrombotic microangiopathy.

Sex	female	male	p
n (%)	15 (44.1)	19 (55.9)	
Mean Age, years (SD)	61.1 (18.9)	57.6 (16.0)	0.51
CD19 count, cells/µl (SD)	86.7 (213.6)	32.8 (106.3)	0.37
Anti-SARS-CoV-2-S1 IgG, BAU/ml (SD)	710.5 (1330.4)	272.8 (774.8)	0.89
IFN-γ-release mIU/ml (SD)	985.3 (940.2)	2213.4 (3177.4)	0.68

Supplemental Table S2. Mean age, CD19 count, SARS-CoV-2-S1-IgGs, and IFN-γ-release is not significantly different between sexes. Abbreviations as in **Table S1**.

Anti-CD20-antibody treatment	regular	individual	p
n (%)	20 (58.8)	14 (41.2)	
Steroid used, n (%)	11 (55.0)	2 (14.3)	
Mean Age, years (SD)	67.4 (11.4)	47.3 (17.4)	0.01
CD19 count, cells/µl (SD)	3.7 (12.1)	132.3 (236.5)	< 0.01
Anti-SARS-CoV-2-S1 IgG, BAU/ml (SD)	11.4 (28.6)	1115.2 (1445.0)	< 0.01
IFN-γ-release mIU/ml (SD)	1722.1 (2398.3)	1833.9 (3038.7)	0.99
**Cause for Immunosuppression**			
AAV, n (%)	20 (100.0)	2 (14.3)	
FSGS, n (%)	0 (0.0)	1 (7.1)	
MGN, n (%)	0 (0.0)	3 (21.4)	
MC, n (%)	0 (0.0)	3 (21.4)	
TMA, n (%)	0 (0.0)	4 (28.6)	
M. Goodpasture, n (%)	0 (0.0)	1 (7.1)	

Supplemental Table S3. CD19-counts and SARS-CoV-2-S1-IgGs are significantly lower in regular vs individual anti-CD19-antibody treatment. Abbreviations: AAV, ANCA-associated vasculitis; FSGS, focal segmental glomerulosclerosis; MGN, membranous glomerulonephritis; MC, minimal change disease; TMA, thrombotic microangiopathy.

Renal immune disease	AAV	non-AAV	p
n (%)	22 (64.7)	12 (35.3)	
Mean Age, years (SD)	65.9 (13.3)	46.8 (16.9)	< 0.01

Supplemental Table S4. AAV is overrepresented in older patients. Abbreviations: AAV, ANCA-associated vasculitis. Abbreviations as in **Table S1**.

Steroid treatment	no additional steroid	additional steroid	p
n (%)	9 (45.0)	11 (55.0)	
Mean Age, years (SD)	67.8 (9.9)	67.1 (12.9)	0.88
Days CD20-Ab to Vaccination (SD)	149.9 (65.6)	119.0 (56.7)	0.33
Days Vaccination to test (SD)	31.2 (27.8)	38.3 (34.1)	0.70
CD19 count, cells/µl (SD)	8.0 (17.5)	0.1 (0.3)	0.02
Anti-SARS-CoV-2-S1 IgG, BAU/ml (SD)	20.7 (41.0)	3.7 (8.1)	0.15
IFN-γ-release mIU/ml (SD	2814.9 (2729.6)	265.2 (258.8)	0.01

Supplemental Table S5. Steroid treatment is associated with impaired humoral and cellular vaccination response in Patients with regular Rituximab treatment. Abbreviations as in **Table S1**.

Supplemental Methods

Quantification of B-Cell counts:

Peripheral B cell counts were measured by flow cytometry using a Navios cytometer (Beckmann Coulter, Brea, CA, USA). Erythrocyte lysis is performed on whole blood samples with a lyse no-wash reagent (Optilyse B, Beckmann Coulter, Brea, CA, USA) prior to immunostaining. Lymphocyte subpopulations are quantified by immunofluorescence staining, using a PE-Cy7-conjugated anti-CD19-antibody (clone J3-119, IM3628, Beckmann Coulter, Brea, CA, USA) for B cell identification. Absolute B cell counts are calculated by multiplying the relative percentage of $CD19^+$ B cells with the lymphocyte count of the white blood cell differential.

Quantification of SARS-CoV-2-S1 IgGs and SARS-CoV-2-interferon response:

SARS-CoV-2-S1 IgG was determined by Siemens SARS-CoV-2 IgG (sCOV), according to manufacturers' instructions. Test results of SARS-CoV-2-S1-IgGs were re-calculated to WHO Binding Antibody Units (BAU/ml) according to manufacturers' instructions. Quantification values ranged from 0 to 3,270 BAU/ml.

The interferon-γ response to SARS-CoV-2-S1 antigen was quantified using the SARS-CoV-2 IGRA assay (EUROIMMUN, Lübeck, Germany) according to manufacturers' instructions. As interferone assays can be difficult to interpret in cases with altered T-cell activity [1], a double-cut-off strategy was developed, integrating the result of background stimulation [2]. In brief, background IFN-*γ* stimulation was integrated in the interpretation of SARS-CoV-2-S1 antigen Stimulation: In individuals with background stimulation < 100 mIU/ml, a cut-off of ≥ 135 mIU/ml was used to define positive cellular response. In individuals with background stimulation ≥ 100 mIU/ml, a cut-off of ≥ 200 mIU/ml was used to define positive cellular response.

Supplemental References

1. Aubry A, Demey B, François C, Duverlie G, Castelain S, Helle F, Brochot E: **Longitudinal analysis and comparison of six serological assays up to eight months post-COVID-19 diagnosis**. *Journal of clinical medicine* 2021, **10**(9):1815.
2. Huzly D, Panning M, Smely F, Enders M, Komp J, Falcone V, Steinmann D: **Accuracy and real life performance of a novel interferon-γ release assay for the detection of SARS-CoV2 specific T cell response**. *Journal of Clinical Virology* 2022, **148**:105098.
